# Evidence of Associations between Cytokine Genes and Subjective Reports of Sleep Disturbance in Oncology Patients and Their Family Caregivers

**DOI:** 10.1371/journal.pone.0040560

**Published:** 2012-07-23

**Authors:** Christine Miaskowski, Bruce A. Cooper, Anand Dhruva, Laura B. Dunn, Dale J. Langford, Janine K. Cataldo, Christina R. Baggott, John D. Merriman, Marylin Dodd, Kathryn Lee, Claudia West, Steven M. Paul, Bradley E. Aouizerat

**Affiliations:** 1 Department of Physiological Nursing, University of California San Francisco, San Francisco, California, United States of America; 2 Department of Community Health Systems, University of California San Francisco, San Francisco, California, United States of America; 3 Department of Medicine, University of California San Francisco, San Francisco, California, United States of America; 4 Department of Psychiatry, University of California San Francisco, San Francisco, California, United States of America; 5 Department of Family Health Care Nursing, University of California San Francisco, San Francisco, California, United States of America; Rush University Medical Center, United States of America

## Abstract

The purposes of this study were to identify distinct latent classes of individuals based on subjective reports of sleep disturbance; to examine differences in demographic, clinical, and symptom characteristics between the latent classes; and to evaluate for variations in pro- and anti-inflammatory cytokine genes between the latent classes. Among 167 oncology outpatients with breast, prostate, lung, or brain cancer and 85 of their FCs, growth mixture modeling (GMM) was used to identify latent classes of individuals based on General Sleep Disturbance Scale (GSDS) obtained prior to, during, and for four months following completion of radiation therapy. Single nucleotide polymorphisms (SNPs) and haplotypes in candidate cytokine genes were interrogated for differences between the two latent classes. Multiple logistic regression was used to assess the effect of phenotypic and genotypic characteristics on GSDS group membership. Two latent classes were identified: lower sleep disturbance (88.5%) and higher sleep disturbance (11.5%). Participants who were younger and had a lower Karnofsky Performance status score were more likely to be in the higher sleep disturbance class. Variation in two cytokine genes (i.e., IL6, NFKB) predicted latent class membership. Evidence was found for latent classes with distinct sleep disturbance trajectories. Unique genetic markers in cytokine genes may partially explain the interindividual heterogeneity characterizing these trajectories.

## Introduction

Sleep disturbance is a significant problem for oncology patients [1], [2] and their family caregivers (FCs) [3]–[6]. Phenotypic characterization of sleep disturbance has focused primarily on the administration of subjective measures and the dichotomization of samples based on clinically meaningful cutpoints. Findings from primarily cross-sectional studies suggest that between 30% and 50% of patients [1], [7], [8] and FCs [5], [9] report sleep disturbance. Patients [8], [10] and FCs [5], [11] report problems with both the initiation and the maintenance of sleep.

Newer statistical methods, like latent class analysis and growth mixture modeling (GMM), may allow for the characterization of subgroups of individuals with distinct types of sleep disturbance. These approaches can be used to classify patients with chronic medical conditions like cancer, as well as their FCs who experience the stressors associated with caring for someone with a chronic illness and who have chronic medical conditions themselves. However, only two population-based studies were found that characterized individuals into distinct subgroups based on self-reported sleep disturbance [12], [13]. In the first cross-sectional study that evaluated individuals enrolled in a Midwest health plan [12], the subgroups were named: distressed (33.2% who had a single sleep complaint that occurred weekly and emotional distress), transient (25.5% who had a variety of sleep-related symptoms that occurred with different frequencies), difficulty maintaining sleep (21.9% who had insomnia symptoms every night), and comorbid with non-restorative sleep (19.4% who had sleep problems every night and daytime dysfunction). The second study from the Finnish Twin Cohort [13], identified three distinct subgroups of individuals (i.e., good sleepers (48%), average sleepers (40%), poor sleepers (12%)). These sleep disturbance subgroups showed a moderate heritability estimate of 46% which suggests a role for genetic factors in sleep disturbance. These findings suggest that latent class methods can be used to identify distinct sleep disturbance phenotypes. However, the studies’ cross-sectional design did not allow for an evaluation of distinct subgroups of individuals whose sleep disturbance might persist over a period of months or years.

The relationships among sleep, circadian rhythms, and the immune system were the subject of a number of recent reviews [14]–[18]. Equally important, a growing body of evidence suggests that sleep is genetically modulated (for reviews see Cirelli, 2011 [19] and Sehgal and Mignot, 2011 [20]). As noted by Sehgal and Mignot [20], while environmental factors can impact sleep, its genetic regulation is substantiated by the identification of polymorphisms in specific sleep disorders and the existence of familial sleep disorders. However, only a limited number of studies have evaluated for associations between inflammatory cytokine genes and sleep disturbance. In one study that evaluated the association between polymorphisms in interleukin-6 (IL6) and obstructive sleep apnea [21], IL6 (rs2069849) was associated with a decreased risk for obstructive sleep apnea, after adjusting for body mass index assuming an additive model. In addition, recent work from our group found an association between a candidate gene in IL6 (rs4719714) and self-reported sleep disturbance in patients and their FCs at the initiation of the patients’ radiation therapy (RT). Common allele homozygotes reported higher levels of sleep disturbance (p = 0.003) than minor allele carriers [22].

Given the paucity of research on the association between cytokine genes and sleep disturbance, the purposes of this study, using GMM in the previously described sample of patients and FCs [22], were to identify distinct latent classes of individuals based on subjective reports of sleep disturbance from prior to the initiation to four months after completion of RT and to examine differences in demographic, clinical, and symptom characteristics between the latent classes. In addition, variations in a number of pro- and anti-inflammatory cytokine genes were evaluated between the latent classes.

## Methods

### Participants and Settings

This descriptive, correlational study is part of a larger, longitudinal study that evaluated multiple symptoms in both patients who underwent primary or adjuvant RT and their FCs [3], [8], [9], [22], [23]. Patients and their FCs were recruited from two RT departments located in a Comprehensive Cancer Center and a community-based oncology program at the time of the patient’s simulation visit.

Patients were eligible to participate if they: were ≥18 years of age; were scheduled to receive primary or adjuvant RT for one of four cancer diagnoses (i.e., breast, prostate, lung, brain); were able to read, write, and understand English; gave written informed consent; and had a Karnofsky Performance Status (KPS) score of ≥60. Patients were excluded if they had: metastatic disease; more than one cancer diagnosis; or a diagnosed sleep disorder. FCs were eligible to participate if they were an adult (≥18 years of age); were able to read, write, and understand English; gave written informed consent; had a KPS score of ≥60; were living with the patient; and did not have a diagnosed sleep disorder.

### Self-report Instruments

The demographic questionnaire obtained information on age, gender, marital status, education, ethnicity, employment status, and the presence of a number of co-morbid conditions. Medical records were reviewed for disease and treatment information.

The Pittsburgh Sleep Quality Index (PSQI) consists of 19 items designed to assess the quality of sleep in the past month. The global PSQI score is the sum of the seven component scores (i.e., subjective sleep quality, sleep latency, sleep duration, habitual sleep efficiency, sleep disturbances, use of sleeping medication, daytime dysfunction). Each component score ranges from 0 to 3 and the global PSQI score ranges from 0 to 21. Higher global and component scores indicate more severe complaints and a higher level of sleep disturbance. A global PSQI score of >5 indicates a significant level of sleep disturbance [24]. The PSQI has established internal consistency, test-retest reliability, and construct validity [24]–[26]. In this study, the Cronbach’s alpha for the global PSQI score was 0.72 for patients and 0.68 for FCs.

The General Sleep Disturbance Scale (GSDS) consists of 21-items designed to assess the quality of sleep in the past week. Each item was rated on a 0 (never) to 7 (everyday) numeric rating scale (NRS). The GSDS total score is the sum of the seven subscale scores (i.e., quality of sleep, quantity of sleep, sleep onset latency, mid-sleep awakenings, early awakenings, medications for sleep, excessive daytime sleepiness) that can range from 0 (no disturbance) to 147 (extreme sleep disturbance). Each mean subscale score can range from 0 to 7. Higher total and subscale scores indicated higher levels of sleep disturbance. Subscale scores of ≥3 and a GSDS total score of ≥43 indicate a significant level of sleep disturbance [4], [8], [9], [11], [27]. The GSDS has well-established validity and reliability in shift workers, pregnant women, and patients with cancer and HIV [28]–[30]. In the current study, the Cronbach’s alpha for the GSDS total score was 0.84 for patients and 0.79 for FCs.

The Lee Fatigue Scale (LFS) consists of 18 items designed to assess physical fatigue and energy [31]. Each item was rated on a 0 to 10 NRS. Total fatigue and energy scores were calculated as the mean of the 13 fatigue items and the 5 energy items, with higher scores indicating greater fatigue severity and higher levels of energy. Participants were asked to rate each item based on how they felt “right now,” within 30 minutes of awakening (morning fatigue, morning energy), and prior to going to bed (evening fatigue, evening energy). The LFS has been used with healthy individuals [31], [32] and in patients with cancer and HIV [30], [33]–[35]. Cutoff scores of ≥3.2 and ≥5.6 indicated high levels of morning and evening fatigue, respectively [4]. Cutoff scores of ≤6.0 and ≤3.5 indicate low levels of morning and evening energy, respectively. The LFS was chosen for this study because it is relatively short, easy to administer, and has well established validity and reliability. In this study, Cronbach’s alphas for evening and morning fatigue at enrollment were 0.96 and 0.95 for patients and 0.95 and 0.96 for FCs, respectively. Cronbach’s alphas for evening and morning energy were 0.95 and 0.96 for patients and 0.95 and 0.96 for FCs, respectively.

The Center for Epidemiological Studies-Depression scale (CES-D) consists of 20 items selected to represent the major symptoms in the clinical syndrome of depression. Scores can range from 0 to 60, with scores of ≥16 indicating the need for individuals to seek clinical evaluation for major depression. The CES-D has well established concurrent and construct validity [36]–[38]. In the current study, the Cronbach’s alpha for the CES-D was 0.88 for patients and 0.84 for FCs.

The Spielberger State-Trait Anxiety Inventories (STAI-T and STAI-S) consist of 20 items each that are rated from 1 to 4. The scores for each scale are summed and can range from 20 to 80. A higher score indicates greater anxiety. The STAI-T measures an individual’s predisposition to anxiety determined by his/her personality and estimates how a person generally feels. The STAI-S measures an individual’s transitory emotional response to a stressful situation. It evaluates the emotional responses of worry, nervousness, tension, and feelings of apprehension related to how a person feels “right now” in a stressful situation. Cutoff scores of ≥31.8 and ≥32.2 indicate high levels of trait and state anxiety, respectively. The STAI-S and STAI-T inventories have well established criterion and construct validity and internal consistency reliability coefficients [39]–[41]. In the current study, the Cronbach’s alphas for the STAI-T and STAI-S were 0.92 and 0.95 for patients and 0.89 and 0.93 for FCs, respectively.

The Attentional Function Index (AFI) consists of 16-items designed to measure attentional fatigue in patients with cancer. Each item is rated on a 0 to 10 NRS. A mean AFI score was calculated, with higher scores indicating greater capacity to direct attention and, therefore, lower levels of attentional fatigue [42], [43]. Based on a previously conducted analysis of the frequency distributions of AFI scores, attentional fatigue can be grouped into categories of functional status (i.e., patients who score <5.0 functioning poorly and experiencing high levels of attentional fatigue, patients who score 5.0 to 7.5 functioning moderately well and experiencing moderate levels of attentional fatigue, patients who score >7.5 functioning well and experiencing low levels of attentional fatigue [44]. The AFI has established reliability and validity [42]. In the current study, Cronbach’s alpha for the AFI was 0.95 for both patients and FCs.

Occurrence of pain was evaluated using the Brief Pain Inventory [45]. Participants who responded yes to the question of having pain were asked to rate its intensity using 0 (no pain) to 10 (worst pain imaginable) NRS.

### Objective Measure of Sleep Disturbance

Objective data on sleep-wake activity rhythms were obtained by continuous noninvasive monitoring of activity over 48 hours using a wrist motion sensor (Mini Motionlogger Actigraph, Ambulatory Monitoring, Inc., Ardsley, NY) [46]–[48]. Seven sleep/wake and one activity/rest variables were selected that were identified by a National Cancer Institute sponsored conference [2], an expert panel that recommended a standard set of research assessments in insomnia [49], and recently published studies [50], [51]. Wrist actigraphy was validated with EEG measures of sleep and awakenings on men and women with both healthy and disturbed sleep patterns [47]–[49]. It provides continuous motion data using a battery-operated wristwatch-size microprocessor that senses motion with a piezo-electric beam and detects movement in all three axes. The accompanying Action 4 software (Ambulatory Monitoring Inc.) allows analysis of activity and nonactivity as well as automatic scoring of sleep and wake episodes in minutes. Actigraphy scores, calculated using specific algorithms correlate with polysomnography in adults at greater than 90% [48].

**Table 1:** Summary of single nucleotide polymorphisms analyzed for pro- and anti-inflammatory cytokine genes and the growth mixture model analysis for general sleep disturbance scale total score.

Gene	SNP	Position	Chr	MAF	Alleles	Chi Square	p-value	Model
IFNG1	rs2069728	66834051	12	.079	G>A	2.18	.335	A
IFNG1	rs2069727	66834490	12	.411	A>G	1.00	.608	A
IFNG1	rs2069718	66836429	12	.442	C>T	2.16	.340	A
IFNG1	rs1861493	66837463	12	.264	A>G	0.62	.733	A
IFNG1	rs1861494	66837676	12	.279	T>C	0.08	.961	A
IFNG1	rs2069709	66839970	12	.008	G>T	FE	1.000	A
IFNG1	HapA3	n/a				0.62	.733	
IFNG1	HapA5	n/a				1.00	.608	
IFNGR1	rs9376268	137574444	6	.246	G>A	1.47	.479	A
IL1B	rs1071676	106042060	2	.198	G>C	2.23	.328	A
IL1B	rs1143643	106042929	2	.331	G>A	0.21	.902	A
IL1B	rs1143642	106043180	2	.095	C>T	1.98	.371	A
IL1B	rs1143634	106045017	2	.196	C>T	2.33	.312	A
IL1B	rs1143633	106045094	2	.345	G>A	0.25	.883	A
IL1B	rs1143630	106046282	2	.103	C>A	0.37	.831	A
IL1B	rs3917356	106046990	2	.432	A>G	1.04	.594	A
IL1B	rs1143629	106048145	2	.353	T>C	0.82	.663	A
IL1B	rs1143627	106049014	2	.390	T>C	1.45	.485	A
IL1B	rs16944	106049494	2	.380	G>A	1.94	.379	A
IL1B	rs1143623	106050452	2	.248	G>C	2.15	.341	A
IL1B	rs13032029	106055022	2	.428	C>T	1.12	.570	A
IL1B	HapA1	n/a				3.87	.145	
IL1B	HapA3	n/a				FE	.191	
IL1B	HapA4	n/a				0.21	.899	
IL1B	HapA5	n/a				2.44	.296	
IL1B	HapB1	n/a				0.15	.928	
IL1B	HapB7	n/a				2.37	.306	
IL1B	HapB9	n/a				1.06	.588	
IL1B	HapB11	n/a				0.29	.863	
IL1R1	rs949963	96533648	2	.213	G>A	2.39	.302	A
IL1R1	rs2228139	96545511	2	.066	C>G	2.29	.318	A
IL1R1	rs3917320	96556738	2	.068	A>C	FE	.238	A
IL1R1	rs2110726	96558145	2	.333	C>T	1.46	.483	A
IL1R1	rs3917332	96560387	2	.124	T>A	0.83	.659	A
IL1R2	rs4141134	96370336	2	.401	T>C	0.44	.805	A
IL1R2	rs11674595	96374804	2	.233	T>C	0.01	.998	A
IL1R2	rs7570441	96380807	2	.393	G>A	0.60	.740	A
IL1R2	HapA1	n/a				1.83	.400	
IL1R2	HapA2	n/a				1.55	.460	
IL1R2	HapA4	n/a				0.40	.818	
IL2	rs1479923	119096993	4	.302	C>T	2.27	.322	A
IL2	rs2069776	119098582	4	.244	T>C	2.44	.295	A
IL2	rs2069772	119099739	4	.238	A>G	2.43	.297	A
IL2	rs2069777	119103043	4	.054	C>T	FE	1.000	A
IL2	rs2069763	119104088	4	.287	T>G	3.95	.138	A
IL2	HapA1	n/a				1.16	.560	
IL2	HapA2	n/a				2.58	.275	
IL2	HapA3	n/a				2.42	.299	
IL2	HapA5	n/a				2.27	.322	
IL4	rs2243248	127200946	5	.101	T>G	2.83	.243	A
IL4	rs2243250	127201455	5	.260	C>T	1.16	.560	A
IL4	rs2070874	127202011	5	.219	C>T	0.01	.996	A
IL4	rs2227284	127205027	5	.399	C>A	0.64	.725	A
IL4	rs2227282	127205481	5	.401	C>G	0.55	.760	A
IL4	rs2243263	127205601	5	.124	G>C	FE	.817	A
IL4	rs2243266	127206091	5	.203	G>A	0.10	.950	A
IL4	rs2243267	127206188	5	.205	G>C	0.11	.947	A
IL4	rs2243274	127207134	5	.262	G>A	0.58	.748	A
IL4	HapA1	n/a				0.33	.847	
IL4	HapA10	n/a				0.23	.891	
IL6	rs4719714	22643793	7	.196	A>T	1.02	.600	A
**IL6**	**rs2069827**	**22648536**	**7**	**.071**	G>T	**8.54**	**.014**	**A**
IL6	rs1800796	22649326	7	.095	G>C	4.71	.095	A
IL6	rs1800795	22649725	7	.355	C>G	0.56	.755	A
IL6	rs2069835	22650951	7	.066	T>C	FE	.722	A
IL6	rs2066992	22651329	7	.091	G>T	2.97	.227	A
IL6	rs2069840	22651652	7	.308	C>G	3.49	.175	A
IL6	rs1554606	22651787	7	.405	T>G	3.38	.185	A
IL6	rs2069845	22653229	7	.405	G>A	3.38	.185	A
**IL6**	**rs2069849**	**22654236**	**7**	**.039**	C>T	**7.68**	**.021**	**A**
IL6	rs2069861	22654734	7	.083	C>T	FE	.790	A
**IL6**	**rs35610689**	**22656903**	**7**	**.242**	A>G	**FE**	**.004**	**D**
IL6	HapA4	n/a				3.43	.180	
IL6	HapA6	n/a				0.80	.670	
IL8	rs4073	70417508	4	.498	T>A	0.14	.932	A
IL8	rs2227306	70418539	4	.366	C>T	1.86	.394	A
IL8	rs2227543	70419394	4	.374	C>T	2.17	.337	A
IL8	HapA1	n/a				5.13	.077	
IL8	HapA3	n/a				1.86	.394	
IL8	HapA4	n/a				0.14	.932	
IL10	rs3024505	177638230	1	.138	C>T	1.49	.476	A
IL10	rs3024498	177639855	1	.236	A>G	2.40	.302	A
IL10	rs3024496	177640190	1	.459	T>C	0.44	.802	A
IL10	rs1878672	177642039	1	.452	G>C	0.06	.969	A
IL10	rs3024492	177642438	1	.207	A>T	1.96	.375	A
IL10	rs1518111	177642971	1	.267	G>A	2.40	.302	A
IL10	rs1518110	177643187	1	.267	G>T	2.40	.302	A
IL10	rs3024491	177643372	1	.448	T>G	0.17	.919	A
IL10	HapA5	n/a				0.51	.775	
IL10	HapA6	n/a				2.45	.294	
IL10	HapA8	n/a				1.85	.397	
IL10	HapA9	n/a				1.35	.510	
IL13	rs1881457	127184713	5	.192	A>C	2.01	.366	A
IL13	rs1800925	127185113	5	.227	C>T	0.20	.903	A
IL13	rs2069743	127185579	5	.021	A>G	FE	.325	A
IL13	rs1295686	127188147	5	.252	G>A	0.17	.920	A
IL13	rs20541	127188268	5	.174	C>T	1.27	.530	A
IL13	HapA1	n/a				0.10	.950	
IL13	HapA4	n/a				1.11	.574	
IL17A	rs4711998	51881422	6	.293	G>A	0.58	.749	A
IL17A	rs8193036	51881562	6	.255	T>C	4.32	.115	A
IL17A	rs3819024	51881855	6	.374	A>G	0.63	.729	A
IL17A	rs2275913	51882102	6	.345	G>A	2.01	.366	A
IL17A	rs3804513	51884266	6	.027	A>T	0.62	.735	A
IL17A	rs7747909	51885318	6	.225	G>A	2.49	.287	A
NFKB1	rs3774933	103645369	4	.444	T>C	0.00	.999	A
NFKB1	rs170731	103667933	4	.397	T>A	1.82	.404	A
NFKB1	rs17032779	103685279	4	.023	T>C	FE	1.000	A
NFKB1	rs230510	103695201	4	.366	T>A	1.26	.533	A
NFKB1	rs230494	103706005	4	.477	A>G	0.62	.733	A
NFKB1	rs4648016	103708706	4	.017	C>T	FE	1.000	A
NFKB1	rs4648018	103709236	4	.025	G>C	FE	.149	A
NFKB1	rs3774956	103727564	4	.479	C>T	0.52	.770	A
NFKB1	rs10489114	103730426	4	.025	A>G	FE	.149	A
NFKB1	rs4648068	103737343	4	.366	A>G	0.74	.692	A
NFKB1	rs4648095	103746914	4	.052	T>C	FE	.506	A
NFKB1	rs4648110	103752867	4	.205	T>A	2.23	.328	A
NFKB1	rs4648135	103755716	4	.060	A>G	FE	.120	A
**NFKB1**	**rs4648141**	**103755947**	**4**	**.188**	G>A	**12.29**	**.002**	**A**
NFKB1	rs1609798	103756488	4	.337	C>T	0.52	.773	A
NFKB1	HapA1	n/a				1.07	.586	
NFKB1	HapA9	n/a				1.77	.414	
NFKB2	rs12772374	104146901	10	.157	A>G	0.25	.881	A
**NFKB2**	**rs7897947**	**104147701**	**10**	**.229**	T>G	**FE**	**.022**	**D**
NFKB2	rs11574849	104149686	10	.085	G>A	0.76	.684	A
NFKB2	rs1056890	104152760	10	.317	C>T	0.38	.827	A
TNFA	rs2857602	31533378	6	.360	T>C	2.35	.309	A
TNFA	rs1800683	31540071	6	.388	G>A	0.51	.774	A
TNFA	rs2239704	31540141	6	.370	G>T	1.95	.378	A
TNFA	rs2229094	31540556	6	.256	T>C	0.70	.706	A
TNFA	rs1041981	31540784	6	.388	C>A	0.51	.774	A
TNFA	rs1799964	31542308	6	.202	T>C	3.52	.172	A
TNFA	rs1800750	31542963	6	.019	G>A	0.18	.913	A
TNFA	rs1800629	31543031	6	.157	G>A	2.27	.321	A
TNFA	rs1800610	31543827	6	.105	C>T	2.57	.277	A
TNFA	rs3093662	31544189	6	.072	A>G	1.08	.584	A
TNFA	HapA1	n/a				0.74	.692	
TNFA	HapA5	n/a				1.77	.412	
TNFA	HapA8	n/a				2.68	.262	

Abbreviations: A  =  Additive model, Chr  =  chromosome, D  =  Dominant model, Hap  =  haplotype, IFNG  =  interferon gamma, IL  =  interleukin, MAF – minor allele frequency, n/a  =  not applicable, NFKB  =  nuclear factor kappa beta, R  =  Recessive model, SNP  =  single nucleotide polymorphism, TNFA  =  tumor necrosis factor alpha.

### Study Procedures

The study was approved by the Committee on Human Research at the University of California, San Francisco and at the second site. Approximately one week prior to the start of RT (i.e., simulation visit when the measurements for RT are made), patients were invited to participate in the study. If the FC was present, a research nurse explained the study protocol to both the patient and FC, determined eligibility, and obtained written informed consent. FCs who were not present were contacted by phone to determine their interest in participation. These FCs completed the enrollment procedures at home.

At the time of the simulation visit, participants completed the self-report questionnaires. Participants completed the symptom questionnaires at 4 weeks after the initiation of RT, at the end of RT, and at 4, 8, 12, and 16 weeks after the completion of RT (i.e., 7 assessments over 6 months) In addition, patients’ medical records were reviewed for disease and treatment information.

At each of the seven assessments, participants completed the LFS [31] before going to bed each night (i.e., evening fatigue, evening energy) and upon arising each morning (i.e., morning fatigue, morning energy) for 2 consecutive days. Participants wore the wrist actigraph to monitor nocturnal sleep/rest and daytime wake/activity continuously for two consecutive weekdays and completed a two day diary. Participants were asked to use the event marker on the wrist actigraph to indicate “lights out” and “lights on” time. Participants reported no difficulties wearing the wrist actigraph. Because the actual time is important in the calculation of the amount of sleep obtained in the amount of time designated for sleep, having an additional source of information about nap times, bed times, and wake times is important. This information was recorded in a two day diary. Upon awakening, the participants used the diary to indicate the number of awakenings during the night.

**Figure 1:**
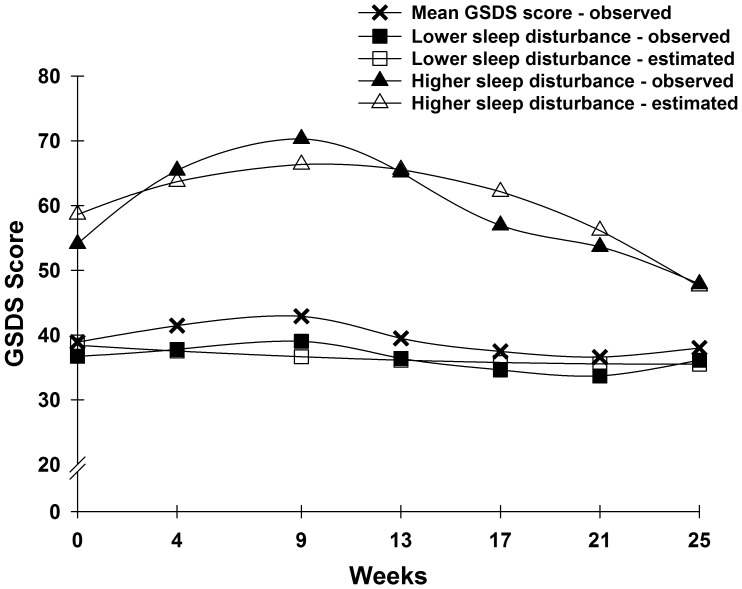
Observed and estimated General Sleep Disturbance Scale (GSDS) trajectories for participants in each of the latent classes, as well as the mean GSDS scores for the total sample.

### Methods of Analysis for Clinical Data

Data were analyzed using SPSS Version 18 [52] and Mplus Version 6.11 [53]. Descriptive statistics and frequency distributions were generated on the sample characteristics and symptom severity scores. Independent sample t-tests and Chi-square analyses were done to evaluate for differences in demographic, clinical and genotypic characteristics between patients and FCs, and between the GMM latent classes.

Actigraphy files in zero-crossing mode, with 30 second intervals, were analyzed using the Cole-Kripke Algorithm in the Action 4 software (Ambulatory Monitoring Inc) by two of the researchers (KL and CW). First, the file was scanned for missing data. Time limits were set for the 48-hour period. The file was reviewed and intervals were individually set for each day and night period using in order of priority as decision guides: the event marker, diary data, channel data, and cascading movement data.

GMM with robust maximum likelihood estimation was used to identify latent classes (i.e., subgroups of participants) with distinct sleep disturbance trajectories (i.e., total GSDS scores) over the 6 months of the study [54]. Because 65% of the participants were in patient-caregiver dyads, models were estimated with “dyad” as a clustering variable, to ensure that any dependency between the GSDS scores for patients and FCs in the same dyad were “controlled for” in the GMM analysis.

The GMM methods are described in detail elsewhere [23]. In brief, a single growth curve that represented the “average” change trajectory was estimated for the total sample. Then the number of latent growth classes that best fit the data was identified using guidelines recommended by a number of experts [55]–[57].

**Table 2:** Fit indices for general sleep disturbance scale gmm solutions over seven assessments, with dyad as a clustering variable.

GMM	LL	AIC	BIC	Entropy	VLMR^c^
1-Class^a^	−6238.023	12508.047	12564.581	n/a	n/a
2-Class^b^	−6208.505	12463.011	12544.279	0.856	59.036^**^
3-Class	−6193.223	12444.445	12546.914	0.811	30.565^n.s.^

* p<.05, p^**^ <.01, ^***^p<.001, n.s.  =  p>.05.

a Random coefficients latent growth curve model with linear and quadratic components; Chi^2^ = 108.81, 26 df, p<0.001, CFI = 0.921, RMSEA = 0.112.

b 2-class model was selected, based on its having the smallest BIC, the largest entropy, and a significant VLMR. Further, the VLMR is not significant for the 3-class model, and the 3-class model estimated a class with only 4% of the sample – a class size that is unlikely to be reliable.

c This value is the Chi^2^ statistic for the VLMR. When significant, the VLMR test provides evidence that the K-class model fits the data better than the K-1-class model.

Abbreviations: GMM  =  Growth mixture model; LL  =  log likelihood; AIC  =  Akaike Information Criteria; BIC  =  Bayesian Information Criterion; VLMR  =  Vuong-Lo-Mendell-Rubin likelihood ratio test; CFI  =  comparative fit index; RMSEA  =  root mean square error of approximation.

First, a model with two latent classes was fit to the data, then a model with three latent classes was fit. The process was repeated until the model with a greater number of classes was not supported. Model fit for the GMM was assessed statistically by identifying the model with the lowest Bayesian Information Criterion (BIC), and by testing the “K” versus “K-1″ class models to determine whether a model with K classes fit the data better than a model with K-1 classes with the Vuong-Lo-Mendell-Rubin likelihood ratio test (VLMR) [55], [56].

The third index used to evaluate model fit was entropy (i.e., the proportion of latent versus predicted class membership. It was estimated for each solution with >.80 being preferred. Better fitting models should produce higher entropy values, indicating consistency between the latent and predicted class membership [53], [58]. In addition to evaluating the fit indices, the best fitting model was visually inspected by plotting observed against model-predicted values to determine whether the predicted trajectories followed the empiric trajectories for the classes, and to evaluate whether the predicted plots “made sense” theoretically and clinically [54].

Intercepts and linear and quadratic slopes for each class were estimated for each model. Intercept variances were estimated for each class and were allowed to differ across classes. Given the relatively small sample size, the within-class quadratic slope variance was fixed at zero, because the model could not be estimated due to a non-positive definite covariance matrix. Mixture models are known to produce solutions at local maxima, so each model was fit with several hundred random starts to be sure that the solution for the model with the maximum log likelihood values was replicated [53]. Missing data for the sleep disturbance scores were accommodated in MPlus 6.11 through the use of Full Information Maximum Likelihood and the use of the Expectation-Maximization algorithm. This method assumes that any missing data are ignorable (i.e., missing at random) [59], [60].

**Table 3:** GMM parameter estimates for general sleep disturbance scale latent class^a^ solution with 7 assessments, with dyad as a clustering variable.

Parameter Estimates^b^	Lower Sleep Disturbance (0) n = 224 (88.5%)	Higher Sleep Disturbance (1) n = 29 (11.5%)
Means	Mean (S.E.)	
Intercept	38.416^***^ (1.777)	58.636^***^ (4.946)
Linear slope	−1.002 (0.708)	6.314^**^ (2.464)
Quadratic slope	0.115 (0.099)	−1.247^***^ (0.296)
Variances		
Intercept	212.766^***^ (31.235)	66.583 (35.399)
Linear Slope	1.051 (0.540)	8.544^**^ (3.313)

* p<.05, **p<.01, ^***^p<.001.

a Trajectory group sizes are for classification of individuals based on their most likely latent class probabilities.

b Growth mixture model estimates were obtained with robust maximum likelihood, with dyad as a clustering variable to account for dependency between patients and caregivers within the same dyad. Quadratic slope variances were fixed at zero to improve estimation.

Abbreviations: GMM  =  Growth mixture model; S.E.  =  standard error.

Adjustments were not made for missing data in comparisons of the classes identified with the GMM. Therefore, the cohort for each analysis was dependent on the largest set of available data across groups. Differences in demographic and clinical characteristics were considered statistically significant at the p<.05 level.

### Methods of Analysis for Genomic Data

#### Gene Selection

Cytokines and their receptors are classes of polypeptides that exercise a major influence on the inflammatory process. These polypeptides are divided into pro- and anti-inflammatory cytokines. Pro-inflammatory cytokines promote systemic inflammation and include: interferon gamma 1 (IFNG1) IFNG receptor 1 (IFNGR1), IL1R1, IL2, IL8, IL17A, nuclear factor kappa beta (NFKB1), NFKB2, and TNFA. Anti-inflammatory cytokines suppress the activity of pro-inflammatory cytokines and include: IL1R2, IL4, IL10, and IL13. Of note, IFNG1, IL1B, and IL6 possess pro- and anti-inflammatory functions [61].

#### Blood collection and genotyping

Genomic DNA was extracted from archived buffy coats maintained by the UCSF Genomic Markers of Symptoms Tissue Bank using the PUREGene DNA Isolation System (Invitrogen, Carlsbad, CA). Of the 287 participants recruited, DNA could be recovered from the archived buffy coats of 253 (i.e., 168 patients and 85 FCs). No differences were found in any demographic and clinical characteristics between participants who did and did not choose to participate in the study or in those participants for whom DNA could not be recovered from archived specimens.

Genotyping was performed blinded to clinical status and positive and negative controls were included. DNA samples were quantitated with a Nanodrop Spectrophotometer (ND-1000) and normalized to a concentration of 50 ng/µL (diluted in 10 mM Tris/1 mM EDTA). Samples were genotyped using the GoldenGate genotyping platform (Illumina, San Diego, CA) and processed according to the standard protocol using GenomeStudio (Illumina, San Diego, CA). Signal intensity profiles and resulting genotype calls for each SNP were visually inspected by two blinded reviewers. Disagreements were adjudicated by a third reviewer.

#### SNP Selection

A combination of tagging SNPs and literature driven SNPs (i.e., SNPs reported as being associated with altered function and/or symptoms) were selected for analysis. Tagging SNPs were required to be common (defined as having a minor allele frequency ≥.05) in public databases (e.g., HapMap). In order to ensure robust genetic association analyses, quality control filtering of SNPs was performed. SNPs with call rates of <95% or Hardy-Weinberg p-values of <.001 were excluded.

As shown in Table 1, a total of 104 SNPs among the 15 candidate genes (IFNG1∶6 SNPs, IFNGR1∶1 SNP; IL1B: 12 SNPs; IL1R1∶5 SNPs; IL1R2∶3 SNPs; IL2∶5 SNPs; IL4∶9 SNPs; IL6∶12 SNPs; IL8∶3 SNPs; IL10∶8 SNPs; IL13∶5 SNPs; IL17A: 6 SNPs; NFKB1∶15 SNPs; NFKB2∶4 SNPs; TNFA: 10 SNPs) passed all quality control filters and were included in the genetic association analyses. Potential functional roles of SNPs associated with specific symptoms were examined using PUPASuite 2.0 [62], a comprehensive search engine that tests a series of functional effects (i.e., non-synonymous changes, altered transcription factor binding sites, exonic splicing enhancing or silencing, splice site alterations, microRNA target alterations).

#### Statistical Analyses

Allele and genotype frequencies were determined by gene counting. Hardy-Weinberg equilibrium was assessed by the Chi-square exact test. Measures of linkage disequilibrium (i.e., D’ and r^2^) were computed from the participants’ genotypes with Haploview 4.2. LD-based haplotype block definition was based on the D’ confidence interval method [63].

For SNPs that were members of the same haploblock, haplotype analyses were conducted in order to localize the association signal within each gene and to determine if haplotypes improved the strength of the association with the phenotype. Haplotypes were constructed using the program PHASE version 2.1 [64]. In order to improve the stability of haplotype inference, the haplotype construction procedure was repeated five times using different seed numbers with each cycle. Only haplotypes that were inferred with probability estimates of ≥85 across the five iterations were retained for downstream analyses. Only inferred haplotypes that occurred with a frequency estimate of ≥15% were included in the association analyses, assuming a dosage model (i.e., analogous to the additive model).

For association tests, three genetic models were assessed for each SNP: additive, dominant, and recessive. Barring trivial improvements (i.e., delta <10%), the genetic model that best fit the data, by maximizing the significance of the p-value was selected for each SNP. Logistic regression analysis that controlled for significant covariates, as well as race/ethnicity, was used to evaluate the association between genotype and pain group membership. Only those genetic associations identified as significant from the univariate analyses were evaluated in the multivariate analyses. A backwards stepwise approach was used to create the most parsimonious model. Except for race/ethnicity, only predictors with a p-value of <0.05 were retained in the final model. Genetic model fit and both unadjusted and covariate-adjusted odds ratios were estimated using the STATA software package, version 9. Based on the recommendations of Rothman [65], adjustments were not made for multiple testing. However, rigorous controls were imposed on the analysis of the SNPs with p-values of <.05. As described above, each of these SNPs was evaluated using logistic regression analyses that controlled for differences in phenotypic characteristics, as well as potential confounding due to population stratification. Only those SNPs that remained significant were included in the final presentation of the results. In addition, the actual number of independent tests is more appropriately considered in relationship to the total number of cytokine genes evaluated (n = 15), because the majority of the SNPs within each gene locus were in linkage disequilibrium. Therefore, the finding of two significant associations is unlikely to be due solely to chance. Findings are reported for all of the SNPs that were evaluated to have these data available in the literature for subsequent comparisons.

Ancestry informative markers (AIMs) can be used as a tool to minimize confounding due to population stratification in case-control association studies [66]–[68]. Homogeneity in ancestry among participants was verified by principal component analysis (PCA) [69], using HelixTree (GoldenHelix, Bozeman, MT). Briefly, the number of principal components (PCs) was sought that distinguished the major racial/ethnic groups in the sample by visual inspection of scatter plots of orthogonal PCs (i.e., PC 1 versus PC2, PC2 versus PC3). This procedure was repeated until no discernible clustering of participants by their self-reported race/ethnicity was possible (data not shown). The first three PCs were selected to adjust for potential confounding due to population substructure (i.e., race/ethnicity) by including them in all logistic regression models (described in the preceding paragraph). One hundred and six ancestry informative markers were included in the analysis.

**Table 4:** Differences in Baseline Demographic and Clinical Characteristics Between the Two Latent Classes for General Sleep Disturbance Scale Total Score.

Characteristic	Lower Sleep Disturbance 224 (88.5%)	Higher Sleep Disturbance29 (11.5%)	p-value
	Mean (SD)	Mean (SD)	
**Age (years)**	**62.1 (11.1)**	**56.4 (11.8)**	**.01**
Education (years)	16.0 (3.0)	15.9 (3.0)	NS
Number of comorbid conditions	4.5 (2.7)	5.4 (2.8)	NS
Weight (pounds)	173.5 (38.1)	188.2 (42.4)	NS
**Karnofsky Performance Status score**	**92.9 (10.9)**	**85.2 (13.5)**	**.001**
	n (%)	n (%)	
Gender (% female)	123 (54.9)	13 (44.8)	NS
Ethnicity			NS
White	168 (75.3)	20 (69.0)	
Asian/Pacific Islander	13 (5.8)	3 (10.3)	
Black	30 (13.5)	4 (13.8)	
Hispanic/Mixed background/Other	12 (5.4)	2 (6.9)	
Lives alone (% yes)	46 (33.1)	8 (27.6)	NS
Married or partnered (% yes)	158 (71.2)	16 (55.2)	NS
Children at home (% yes)	30 (16.0)	6 (25.0)	NS
Older adult at home (% yes)	6 (3.2)	1 (4.2)	NS
Work for pay (% yes)	103 (46.8)	12 (42.9)	NS
**Patient/Family caregiver (% Patient)**	**139 (62.1)**	**29 (100.0)**	**<.001**

## Results

### Participant Characteristics

The majority of the participants were Caucasian (74.7%), well educated (15.9 (±3.0) years), and married/partnered (69.3%). The mean age of the total sample was 61.5 (±11.3) years. The average participant had over four comorbid conditions (4.6 (±2.7) and a mean KPS score of 92.0 (±11.5). Gender was evenly represented within the total sample with 46.2% male and 53.8% female participants. Patients made up 66.4% of the total sample. Approximately 38% of the patients had breast cancer, 49% had prostate cancer, 7% had brain cancer, and 6% had lung cancer. The majority of the FCs (91%) was the patients’ spouses.

No significant differences were found between patients and FCs in age (60.9 (±11.6) years versus 62.5 (±10.5) years), KPS score (91.1 (±11.9) versus 93.7 (±10.6)), and number of comorbidities (4.8 (±2.6) versus 4.2 (±2.9)). In addition, at the time of enrollment, no significant differences were found between patients and FCs in their ratings of worst pain (2.0 (±3.2) versus 1.5 (±3.1)), fatigue (4.2 (±2.0) versus 4.5 (±2.0)), sleep disturbance (38.9 (±19.6) versus 38.7 (±16.7)), and depression (9.1 (±8.7) versus 8.3 (±7.2)).

### Results of GMM Analysis

Two distinct latent classes of GSDS trajectories were identified using GMM (Figure 1). The fit indices for the various models are shown in Table 2. A two-class model was selected because its BIC was smaller than the one-class and three-class models. In addition, each class in the two-class model had a reasonable size and interpretability [55].

The parameter estimates for the two latent classes are listed in Table 3. The largest percentage of participants was classified into the lower sleep disturbance class (88.5%). These participants had GSDS scores of 38.4 at enrollment, with a stable trajectory over the course of the study. Participants in the higher sleep disturbance class (11.5%) had a mean GSDS score of 58.6 that increased and then decreased slightly over the course of the study. The terms “lower” and “higher” are used to describe these two latent classes because the mean GSDS scores across the six months of the study for participants in the lower class approached the clinically meaningful cutoff of ≥43.

### Examination of Possible Patient/FC Status Effects and Dyadic Effects

No significant differences were found in patients’ (38.9 (±19.6)) and FCs’ (38.7 (±16.7), p = .904) mean GSDS scores at enrollment. However, a significantly higher percentage of participants in the higher sleep disturbance class were patients (100%) than in the lower sleep disturbance class (62.1%, p<.001; Table 4). After taking patient and FC dependency within dyads into account in the GMM analyses, significant differences in linear and quadratic slopes (both p<.001) were found between patients and FCs for the two-class GMM solution. However, this difference was due entirely to the strong positive trajectory for the smaller class. No differences were found in linear and quadratic slopes between patients and FCs within the larger class. Intercepts did not differ between patients and FCs.

### Differences in Demographic and Clinical Characteristics

As summarized in Table 4, no differences were found between the two latent classes in gender, ethnicity, education, employment status, living arrangements, having children living at home, having an older adult at home, number of comorbid conditions, and weight. However, participants in the higher sleep disturbance class were more likely to be younger (p = .01) and have a lower KPS score (p = .001).

### Differences in Symptom Characteristics

As summarized in Table 5, significant differences were found between the two latent classes in the majority of the symptoms assessed prior to the initiation of RT. For those symptom scores with significant between group differences, participants in the higher sleep disturbance class reported higher symptom severity scores than participants in the lower sleep disturbance class.

**Table 5:** Differences in Baseline Symptom Severity Scores Between the Two Latent Classes for General Sleep Disturbance Scale Total Score.

Characteristic	Lower Sleep Disturbance 224 (88.5%)	Higher Sleep Disturbance 29 (11.5%)	Statistics
	Mean (SD)	Mean (SD)	
**Psychological Symptoms at Baseline**			
**STAI-S**	**30.4 (10.9)**	**35.1 (9.2)**	**.029**
**STAI-T**	**33.5 (9.9)**	**38.3 (9.0)**	**.014**
**CES-D Total**	**8.0 (7.9)**	**14.9 (7.7)**	**<.001**
**Pittsburgh Sleep Quality Index (PSQI) Scores at Baseline**			
**Subjective sleep quality**	**0.9 (0.7)**	**1.4 (0.7)**	**<.001**
**Sleep latency**	**0.9 (0.9)**	**1.4 (1.0)**	**.008**
**Sleep duration**	**0.9 (0.9)**	**1.4 (1.0)**	**.003**
Habitual sleep efficiency	0.7 (1.0)	0.9 (1.0)	NS
**Sleep disturbance**	**1.3 (0.5)**	**1.8 (0.6)**	**<.001**
Use of sleeping medication	0.6 (1.1)	0.9 (1.3)	NS
**Daytime dysfunction**	**0.7 (0.6)**	**1.2 (0.7)**	**<.001**
**PSQI Global score**	**5.9 (3.5)**	**8.9 (3.4)**	**<.001**
**General Sleep Disturbance Scale Scores at Baseline**			
**Quality**	**2.3 (1.7)**	**3.8 (1.9)**	**<.001**
**Sleep onset latency**	**1.4 (2.0)**	**2.3 (2.0)**	**.025**
**Quantity**	**4.3 (1.3)**	**5.1 (1.2)**	**.001**
Sleep medication	0.3 (0.6)	0.4 (0.6)	NS
**Mid-sleep awakenings**	**4.3 (2.6)**	**5.7 (1.9)**	**.001**
**Early awakenings**	**2.1 (2.2)**	**3.7 (2.2)**	**<.001**
**Excessive daytime sleepiness**	**1.7 (1.3)**	**2.7 (1.3)**	**<.001**
**Total GSDS score**	**36.7 (17.7)**	**55.0 (17.8)**	**<.001**
**Actigraphy Parameters at Baseline**			
Sleep period time (minutes)	485.5 (75.2)	476.2 (72.1)	NS
Total sleep time (minutes)	403.2 (79.6)	372.8 (106.5)	NS
**Sleep efficiency**	**83.1 (11.9)**	**77.7 (18.4)**	**.037**
**Wake after sleep onset (% of TST)**	**13.3 (11.1)**	**18.4 (17.4)**	**.037**
Wake number	16.9 (8.8)	15.5 (8.9)	NS
Wake duration (minutes)	3.7 (2.4)	7.2 (12.0)	NS
Sleep onset latency (minutes)	13.6 (11.9)	23.7 (42.1)	NS
% Daytime sleep	6.6 (10.8)	10.3 (20.0)	NS
**Fatigue and Energy Scores at Baseline**			
**Evening fatigue**	**4.1 (2.1)**	**5.5 (1.3)**	**<.001**
**Morning fatigue**	**2.1 (1.9)**	**3.8 (1.8)**	**<.001**
Evening energy	4.5 (1.8)	3.9 (1.8)	NS
**Morning energy**	**6.0 (2.0)**	**4.8 (1.8)**	**.003**
**Attentional fatigue**	**7.3 (1.7)**	**6.3 (2.0)**	**.005**
	n (%)	n (%)	
**Pain (% yes)**	**93 (41.5)**	**28 (96.6)**	**<.001**

Abbreviations: STAI-S  =  Spielberger State-Trait Anxiety Inventory – State subscale; STAI-T  =  Spielberger State-Trait Anxiety Inventory – Trait subscale; CES-D  =  Center for Epidemiological Studies – Depression scale; GSDS  =  General Sleep Disturbance Scale; TST  =  Total sleep time.

### Candidate Gene Analyses of the Two GMM Classes

As summarized in Table 1, the minor allele frequency was significantly different between the two latent classes for five SNPs: IL6 rs2069827, IL6 rs2069849, IL6 rs35610689, NFKB1 rs4648141, and NFKB2 rs7897947. For IL6 rs2069827 (p = .014) and IL6 rs2069849 (p = .021), an additive model fit the data best. For IL6 rs35610689 (p = .004), a dominant model fit the data best. For NFKB1 rs4648141 (p = .002), an additive model fit the data best. For NFKB2 rs7897947 (p = .022), a dominant model fit the data best. No significant differences were found between the latent classes for any of the haplotypes analyzed.

### Regression Analyses of Candidate Genes and GMM Latent Classes

In order to better estimate the magnitude (i.e., odds ratio, OR) and precision (95% confidence interval, CI) of genotype on sleep disturbance class membership (i.e., lower sleep disturbance, higher sleep disturbance), multivariable logistic regression analyses were performed that included the following variables in the models: genotype, age, functional status, and ethnicity (i.e., White, Black, Asian/Pacific Islander, Hispanic/Mixed ethnic background/other); and three PCs to control for genetic background. Given the fact that FCs were not represented in the higher sleep disturbance class, this variable could not be evaluated in the regression analyses.

**Figure 2:**
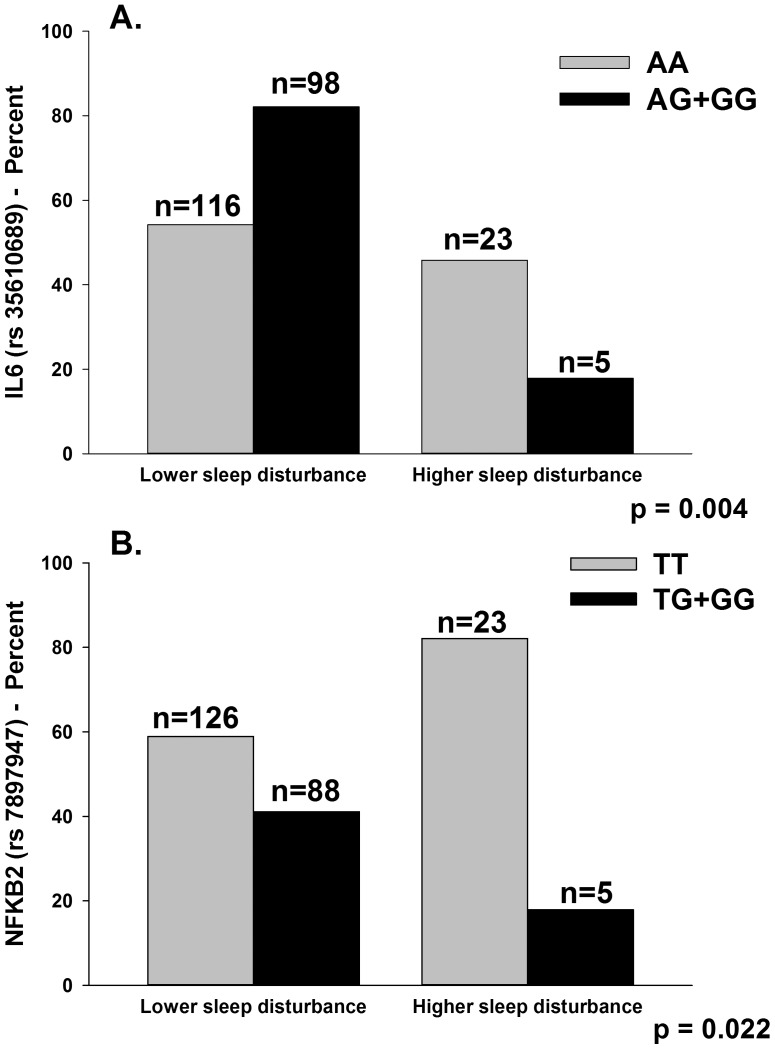
Panel A illustrates differences between the latent classes in the percentages of participants who were homozygous for the common allele (AA) or heterozygous or homozygous for the minor allele (AG+GG) for rs35610689 in interleukin 6 (IL6). Panel B illustrates differences between the latent classes in the percentages of participants who were homozygous for the common allele (TT) or heterozygous or homozygous for the minor allele (TG+GG) for rs7897947 in nuclear factor kappa beta 2 (NFKB2).

The only genetic associations that remained significant in the multivariable logistic regression analyses were for IL6 rs35610689 (Table 6, Figure 2A) and NFKB2 rs7897947 (Table 6, Figure 2B). In the regression analysis for IL6 rs35610689, after controlling for race/ethnicity, genotype and functional status were the only variables retained in the final model (p = .0033). The overall model explained 13.4% of the variance in GMM latent class membership. Controlling for functional status and race/ethnicity, carrying one or two doses of the minor allele (i.e., AG+GG) was associated with a 78% decrease in the odds of belonging to the higher sleep disturbance class (p = .006). Genotype uniquely explained 5.53% of the variance in GMM latent class membership.

**Table 6:** Multiple Logistic Regression Analyses for Interleukin 6 (IL6) rs35610689 and Nuclear Factor Kappa Beta 2 Subunit (NFKB2) rs7897947 to Predict Higher Sleep Disturbance Class.

Growth Mixture ModelClass Comparison	Predictor	Odds Ratio	Standard Error	95% CI	Z	p-value
Lower to Higher SleepDisturbance (n = 235)	IL6 genotype	0.22	0.120	0.076, 0.642	−2.78	.006
	Functional status	0.58	0.092	0.422, 0.790	−3.44	.001
	Overall model fit: χ^2^ = 23.06, p = .0033 R^2^ = 0.1343					
Lower to Higher SleepDisturbance (n = 235)	NFKB2 genotype	0.26	0.139	0.089, 0.742	−2.51	.012
	Functional status	0.59	0.094	0.436, 0.809	−3.31	.001
	Overall model fit: χ^2^ = 21.22, p = .0066 R^2^ = 0.1236					

For each model, the first three principle components identified from the analysis of ancestry informative markers as well as self-report race/ethnicity (White, Asian/Pacific Islander, Black, Hispanic/Mixed background/Other) were retained in all models to adjust for potential confounding due to race or ethnicity (data not shown). Predictors evaluated in the model included genotype (IL6 rs35610689: AA versus AG+GG; NFKB2 rs7897947: TT versus TG + GG), age (5 year increments), and functional status (KPS score, 10 point increments). Patient versus family caregiver (FC) status could not be included in the regression analyses because no FCs were included in the higher sleep disturbance class.

In the regression analysis for NFKB2 rs7898947, after controlling for race/ethnicity, genotype and functional status were the only variables retained in the final model (p = .0066). The overall model explained 12.4% of the variance in GMM latent class membership. Controlling for functional status and race/ethnicity, carrying one or two doses of the minor allele (i.e., TG+GG) was associated with a 74% decrease in the odds of belonging to the higher sleep disturbance class (p = .012). Genotype uniquely explained 4.46% of the variance in GMM latent class membership.

## Discussion

This study is the first to identify distinct subgroups of oncology patients and FCs based on changes in self-reported sleep disturbance and to evaluate associations between these subgroups and cytokine gene variations. While no differences in total GSDS scores, prior to the initiation of RT, were found between patients and FCs, the higher class was composed only of patients. In addition, these individuals were significantly younger and reported a lower functional status that was not only statistically significant but clinically meaningful (effect size, d = 0.67) [70], [71]. Of the 29 patients in the higher class, 44.8% had prostate cancer and 37.9% had breast cancer.

Compared to the lower class, the mean total GSDS score of the higher sleep disturbance class at enrollment represents not only a statistically significant, but a clinically meaningful difference in sleep disturbance score (d = −0.98). Not surprisingly, participants in the higher class reported significantly higher scores on all of the PSQI and GSDS subscales, except for use of sleep medications. While specific studies on sleep medication use in oncology patients and FCs were not identified, previous cross-sectional studies that used either the PSQI or GSDS to evaluate sleep disturbance in oncology patients [26], [35], [50], [72]–[74] and their FCs [75] found similar subscale and total scores, as well as the lack of use of sleep medications. Reasons for the low rates of sleep medication use may relate to under assessment or under-reporting of sleep disturbance in both oncology patients and their FCs. Alternatively, patients and FCs may choose not to take sleep medications because of concerns about physical dependence or side effects.

An interesting finding related to the sleep disturbance phenotype identified in this study was that, except for sleep efficiency and wake after sleep onset, no between class differences were found in any of the objective sleep parameters. On average, participants in both classes slept approximately 6 to 6.5 hours per night. While significantly different, both classes of participants had sleep efficiency scores below the desired 95% that characterizes a good night’s sleep. In fact, an examination of both the subjective scores and objective parameters suggest that both classes of participants had significant amounts of sleep disturbance. For example, participants in the lower class reported problems with sleep quantity and mid-sleep awakenings on more than three days per week. An average of 15 to 16 awakenings per night identified using actigraphy provides additional evidence that all of these participants had problems with sleep maintenance.

Consistent with previous reports [50], [51], participants in the higher class reported higher levels of morning and evening fatigue, lower levels of morning energy, and worse attentional fatigue at the time of enrollment into the study. In addition, almost all of the participants in the higher class reported the occurrence of pain, while less than half of the participants in the lower class reported pain. Finally, higher depression and anxiety scores were reported by participants in the higher class. Taken together, these findings suggest that participants with higher levels of sleep disturbance need to be assessed for the presence of other concurrent symptoms. Future studies are warranted that evaluate for changes in the relationships among these symptoms over time. These types of studies may identify the primary symptom that drives the severity of the other symptoms and may suggest an underlying mechanism for one or more symptoms as well as more targeted interventions.

While initial univariate analyses found between class differences in a number of SNPs for IL6, NFKB1, and NFKB2, after controlling for significant covariates and race/ethnicity, only two SNPs explained a significant amount of the variance in latent class membership. For both NFKB2 (rs7897947) and IL6 (rs35610689), carriers of one or two doses of the minor allele was associated with a decrease in the odds (i.e., 74%, 78%, respectively) of belonging to the higher sleep disturbance class. Each SNP explained a significant amount of the variance in GMM latent class membership (i.e., 5.5% and 4.5%, respectively). Both of these SNPs are located in introns. While the function of these polymorphisms is unknown, they may be surrogates for unmeasured functional polymorphisms that are in linkage disequilibrium with these SNPs.

NFKB is a generic name for an evolutionarily conserved transcription factor system that contributes to the effective mounting of an immune response as well as to the regulation of cell proliferation, development, and apoptosis. The NFKB system appears to be activated in stressful situations and in response to tissue damage [76]. In addition, NFKB is involved in the regulation of chemokine and cytokine genes. For example, work by Libermann and Baltimore [77] determined through in vitro studies that NFKB is an important mediator for the activation of the IL6 gene by a variety of IL6 inducers.

While no studies were found that demonstrated an association between NFKB and sleep disturbance, three studies have identified associations between IL6 and sleep disturbance [21], [22], [78]. In a study that compared patients with chronic low back pain to age- and sex-matched controls [78], poorer sleep quality was associated with higher levels of IL6 in the patients with chronic low back pain. In another study [21], a synonymous SNP within the IL6 coding region (rs2069849) was protective against obstructive sleep apnea in a sample of African Americans and produced qualitatively similar, albeit nonsignificant findings in a replication cohort of European-Americans due to a relatively low minor allele frequency. Interestingly, this same SNP (IL6 rs2069849, p = .014) was significant in the univariate analyses in our GMM study that consisted primarily of Caucasian participants. Additional research is warranted to evaluate the relationship between this SNP (IL6 rs2069849) and a variety of sleep disturbances and sleep disorders.

It is interesting to note that the SNP in IL6 (rs4719714), which was not significant in this study, was associated with higher levels of sleep disturbance in this same sample of patients and FCs at the time of the patients’ initiation of RT [22]. This finding suggests that the methods used to characterize the sleep disturbance phenotype influence the outcomes of candidate gene studies. The functional significance of the NFKB2 (rs7897947) and IL6 (rs35610689) SNPs that were associated with a decreased odds of belonging to the higher sleep disturbance class remain to be determined. Given the relatively small sample size, neither cumulative nor interaction effects between these two SNPs were evaluated. Future studies are warranted to replicate these findings and to evaluate for gene x gene as well as gene x environment interactions.

Several study limitations need to be acknowledged. While the sample size for the GMM analysis was adequate [56], [57], larger samples may identify additional latent classes. In addition, findings from this study must be interpreted with caution until they are replicated in future studies. Ideally future studies should be done with sample sizes that are large enough to allow for confirmatory analyses of both the number and trajectories of the latent classes, as well as the phenotypic and genotypic characteristics that are unique to each class. In terms of the genetic analyses, additional studies with larger samples are needed to confirm the associations found in this study. Future studies can evaluate additional cytokine SNPs as well as serum levels of these cytokines to obtain more information on the functional significance of these genetic variations. In addition, future studies can examine the associations between different sleep disturbance phenotypes or endophenotypes (e.g., daytime sleepiness, total sleep time), using both subjective and objective measures, and pro- and anti-inflammatory cytokine genes.

Despite these limitations, findings from this study provide preliminary evidence for distinct sleep disturbance phenotypes in oncology patients and their FCs. Because the higher risk phenotype was associated with higher levels of depression and anxiety, as well as higher levels of physical and attentional fatigue, clinicians need to assess for multiple co-occurring symptoms in both oncology patients and their FCs. Finally, the candidate gene associations found in this study suggest a role for inflammation in the development of persistent levels of sleep disturbance.
